# Supplementary material for: Prevalence and clinical implications of respiratory viruses in stable chronic obstructive pulmonary disease (COPD) and exacerbations: a systematic review and meta-analysis protocol
Source: BMJ Open. 2020 Apr 7;10(4):e035640. doi: 10.1136/bmjopen-2019-035640 (PMC7170624; doi:10.1136/bmjopen-2019-035640)
Supplement: Supplementary data [file bmjopen-2019-035640supp001.pdf]

Online Appendix

Title: Prevalence and clinical implications of respiratory viruses in stable chronic obstructive pulmonary disease (COPD) and exacerbations: A systematic review and meta-analysis protocol.

Authors: Anastasia M. Kefala, Rebecca Fortescue, Gioulinta S. Alimani, Prodromos Kanavidis, Melissa Jane McDonnell, Emmanouil Magiorkinis, Dimitrios Paraskevis, Chrysa Voyiatzaki, Georgios A. Mathioudakis, Nikolaos G. Papadopoulos, Jørgen Vestbo, Apostolos Beloukas, Alexander G. Mathioudakis.

Search Strategy - Medline, PubMed & Cochrane Library

#1	Chronic Obstructive Pulmonary Disease [MH]
#2	Lung Diseases, Obstructive [MH:NOEXP]
#3	Emphysema [MH]
#4	Chronic Bronchitis [MH]
#5	COPD [tiab]
#6	COAD [tiab]
#7	"Chronic Bronchitis" [tiab]
#8	Emphysema [tiab]
#9	Obstructive[ti]
#10	(Pulmonary OR Respiratory OR Airway OR Airflow OR Lung)[ti]
#11	#9 AND #10
#12	AECOPD[tiab]
#13	IECOPD[tiab]
#14	#1 OR #2 OR #3 OR #4 OR #5 OR #6 OR #7 OR #8 OR #11 OR #12 OR #13
#15	Viruses[MH]
#16	Influenza, Human[MH]
#17	Rhinovirus[MH]
#18	Respiratory Syncytial Viruses[MH]
#19	Coronavirus[MH]
#20	Paramyxoviridae Infections[MH]
#21	Orthomyxoviridae[MH]
#22	Adenoviridae [MH]

#23 Picornaviridae[MH]

#24 Metapneumovirus[MH]

#25 Enterovirus[MH]

#26 Cytomegalovirus[MH]

#27 Herpesvirus 3, Human[MH]

#28 Virus*[tiab]

#29 Viral*[tiab]

#30 Influenza*[tiab]

#31 Rhinovir*[tiab]

#32 Respiratory Syncytial Vir*[tiab]

#33 Coronavir*[tiab]

#34 Paramyxovir*[tiab]

#35 Orthomyxovir*[tiab]

#36 Adenovir*[tiab]

#37 Picornavir*[tiab]

#38 Metapneumov*[tiab]

#39 vzv[tiab]

#40 varicella[tiab]

#41 Enterovir*[tiab]

#42 Parainfluenza[tiab]

#43 Echovir*[tiab]

#44 #15 or #16 or #17 or #18 or #19 or #20 or #21 or #22 or #23 or #24 or #25 or #26 or #27 or #28 or #29 or #30 or #31 or #32 or #33 or #34 or #35 or #36 or #37 or #38 or #39 or #40 or #41 or #42 or #43

#45 Animals[mh] not (humans[mh])

#46 (child[mh]or (adolescent[mh])) not (adult[mh])

#47 editorial[pulication type]

#48 review[publication type] not (systematic review [publication type])

#49 #14 and #44

#50 #49 NOT (#45 or #46 or #47 or #48)

Search Strategy: EMBASE

1 exp Chronic Obstructive Pulmonary Disease/

2 exp Lung diseases, obstructive/

3 exp Chronic bronchitis/

4 exp emphysema/

5 COPD.tw.

6 COAD.tw.

7 (chronic adj2 bronchit$).tw.

8 (obstructive adj3 (pulmonary or lung$ or airway$ or airflow$ or bronch$ or respirat$)).tw.

9 AECOPD.tw

10 IECOPD.tw

11 ECOPD.tw

12 1 or 2 or 3 or 4 or 5 or 6 or 7 or 8 or 9 or 10 or 11

13 exp virus/

14 exp influenza/

15 exp rhinovirus/

16 exp Respiratory Syncytial Virus/

17 exp Coronavirus/

18 exp Paramyxoviridae Infections/

19 exp Orthomyxoviridae/

20 exp Adenoviridae/

21 exp Picornaviridae/

22 exp Metapneumovirus/

23 exp Enterovirus/

24 exp Cytomegalovirus/

25 exp bocavirus/

26 virus$.tw.

27 viral$.tw.

28 Influenza$.tw.

29 (("haemophilus influenzae" or "h. influenza") not influenza).tw.

30 28 not 29

31 Rhinovir$.tw.

32 Respiratory Syncytial Vir$.tw.

33 Coronavir$.tw.

34 Paramyxovir$.tw.

35 Orthomyxovir$.tw.

36 Adenovir$.tw.

37 Picornavir$.tw.

38 Metapneumov$.tw.

39 Enterovir$.tw.

40 Parainfluenza.tw.

41 Echovir$.tw.

42 Bocavir$.tw.

43 vzv.tw.

44 varicella.tw.

45 13 or 14 or 15 or 16 or 17 or 18 or 19 or 20 or 21 or 22 or 23 or 24 or 25 or 26 or 27 or 30 or 31 or 32 or 33 or 34 or 35 or 36 or 37 or 38 or 39 or 40 or 41 or 42 or 43 or 44

46 exp animals/ not exp humans/

47 (exp child/ or exp adolescent/) not exp adult/

48 exp editorial/ or (exp review/ not (exp systematic review/))

49 12 and 45

50 49 not (46 or 47 or 48)

Data extraction: Variables to be captured.

Study characteristics - General: study ID, study/cohort name, study registration number, full references of all citations referring to the study, year of publication, country(-ies), continent(s), number of recruiting centres, study design, study setting.

Description of the study population: Stable COPD or exacerbations, general or specific population tested, total number of participants evaluated during stable disease, total number of participants evaluated during exacerbation, age, sex, smoking history, sampling period, %sampled during the flu season [October-May], spirometric severity of COPD, history of exacerbations, respiratory symptoms [mMRC, CAT], use of ICS (%), severity of the index exacerbation, >1 exacerbation allowed per participant.

Risk of bias assessment: The relevant risk of bias tool(s) will be completed for each included study.

Respiratory viruses: Type of sample, number of viruses evaluated, number of patients testing positive for any virus, number of patients tested for any virus, number of patients testing positive for more than one virus, number of patients testing positive for each virus type or subtype tested, number of patients that were tested in each case, data on the viral loads of each virus, assay used (commercial versus in-house), performance characteristics of the assays used.

Clinical outcomes: Data on each of the selected outcome measures will be extracted. These will include a detailed description of the definition of the outcome, the effect estimates and confidence intervals, as well as the timing of every measurement. Outcome measures in relevant subgroups of participants will also be extracted.

Data collected during stable disease versus exacerbations will be clearly separated.
