# Supplementary material for: Prevalence and clinical implications of respiratory viruses in stable chronic obstructive pulmonary disease (COPD) and exacerbations: a systematic review and meta-analysis protocol
Source: BMJ Open. 2020 Apr 7;10(4):e035640. doi: 10.1136/bmjopen-2019-035640 (PMC7170624; doi:10.1136/bmjopen-2019-035640)
Supplement: Author's manuscript [file bmjopen-2019-035640.draft_revisions.pdf]

BMJ Open

BMJ Open is committed to open peer review. As part of this commitment we make the peer review history of every article we publish publicly available.

When an article is published we post the peer reviewers' comments and the authors' responses online. We also post the versions of the paper that were used during peer review. These are the versions that the peer review comments apply to.

The versions of the paper that follow are the versions that were submitted during the peer review process. They are not the versions of record or the final published versions. They should not be cited or distributed as the published version of this manuscript.

BMJ Open is an open access journal and the full, final, typeset and author-corrected version of record of the manuscript is available on our site with no access controls, subscription charges or pay-per-view fees (http://bmjopen.bmj.com).

If you have any questions on BMJ Open's open peer review process please email info.bmjopen@bmj.com

BMJ Open

Prevalence and clinical implications of respiratory viruses in stable chronic obstructive pulmonary disease (COPD) and exacerbations: A systematic review and meta-analysis protocol.

Journal:	*BMJ Open*
Manuscript ID	bmjopen-2019-035640
Article Type:	Protocol
Date Submitted by the Author:	11-Nov-2019

Complete List of Authors:	Kefala, Anastasia; University of West Attica, Department of Biomedical Sciences<br>Fortescue, Rebecca ; University of London Saint George's, Cochrane Airways, Population Health Research Institute<br>Alimani, Gioulinta; Athens Breath Centre; University of West Attica, Department of Biomedical Sciences<br>Kanavidis, Prodromos; National and Kapodistrian University of Athens, First Department of Surgery<br>McDonnell, Melissa Jane; Galway University Hospitals, Department of Respiratory Medicine<br>Magiorkinis, Emmanouil; University of West Attica, Department of Biomedical Sciences; Sotiria Regional Chest Disease Hospital of Athens, Department of Laboratory Haematology<br>Megremis, Spyridon; The University of Manchester, Division of Evolution and Genomic Science<br>Paraskevis, Dimitrios; National and Kapodistrian University of Athens, Department of Hygiene Epidemiology and Medical Statistics<br>Voyiatzaki, Chrysa; University of West Attica, Department of Biomedical Sciences<br>Mathioudakis, Georgios; Athens Breath Centre<br>Papageorgiou, Effie; University of West Attica, Department of Biomedical Sciences<br>Papadopoulos, Nikolaos; The University of Manchester, Division of Infection, Immunity and Respiratory Medicine; National and Kapodistrian University of Athens, Allergy Department, 2nd Paediatric Clinic<br>Vestbo, Joergen; The University of Manchester, Division of Infection, Immunity and Respiratory Medicine; Manchester University NHS Foundation Trust, North West Lung Centre<br>Beloukas, Apostolos; University of West Attica, Department of Biomedical Sciences<br>Mathioudakis, Alexander; The University of Manchester, Division of Infection, Immunity and Respiratory Medicine; Manchester University NHS Foundation Trust, North West Lung Centre

Keywords:	Epidemiology < INFECTIOUS DISEASES, Epidemiology < THORACIC MEDICINE, Chronic airways disease < THORACIC MEDICINE, Adult thoracic medicine < THORACIC MEDICINE, Respiratory infections < THORACIC MEDICINE

I, the Submitting Author has the right to grant and does grant on behalf of all authors of the Work (as defined in the below author licence), an exclusive licence and/or a non-exclusive licence for contributions from authors who are: i) UK Crown employees; ii) where BMJ has agreed a CC-BY licence shall apply, and/or iii) in accordance with the terms applicable for US Federal Government officers or employees acting as part of their official duties; on a worldwide, perpetual, irrevocable, royalty-free basis to BMJ Publishing Group Ltd ("BMJ") its licensees and where the relevant Journal is co-owned by BMJ to the co-owners of the Journal, to publish the Work in this journal and any other BMJ products and to exploit all rights, as set out in our licence.

The Submitting Author accepts and understands that any supply made under these terms is made by BMJ to the Submitting Author unless you are acting as an employee on behalf of your employer or a postgraduate student of an affiliated institution which is paying any applicable article publishing charge ("APC") for Open Access articles. Where the Submitting Author wishes to make the Work available on an Open Access basis (and intends to pay the relevant APC), the terms of reuse of such Open Access shall be governed by a Creative Commons licence – details of these licences and which Creative Commons licence will apply to this Work are set out in our licence referred to above.

Other than as permitted in any relevant BMJ Author's Self Archiving Policies, I confirm this Work has not been accepted for publication elsewhere, is not being considered for publication elsewhere and does not duplicate material already published. I confirm all authors consent to publication of this Work and authorise the granting of this licence.

For peer review only

Title: Prevalence and clinical implications of respiratory viruses in stable chronic obstructive pulmonary disease (COPD) and exacerbations: A systematic review and meta-analysis protocol.

Authors: Anastasia M. Kefala[1], Rebecca Fortescue[2], Gioulinta S. Alimani[1,3], Prodromos Kanavidis[4], Melissa Jane McDonnell[5], Emmanouil Magiorkinis[1,6], Spyridon Megremis[7], Dimitrios Paraskevis[8], Chrysa Voyiatzaki[1], Georgios A. Mathioudakis[3], Effie Papageorgiou[1], Nikolaos G. Papadopoulos[9,10], Joergen Vestbo[9,11], Apostolos Beloukas[1,12#], Alexander G. Mathioudakis[9,11#].

[#] AB and AGM equally shared last authorship to this work.

Affiliations:

[1] Department of Biomedical Sciences, University of West Attica, Athens, Greece.

[2] Cochrane Airways, Population Health Research Institute, St George's, University of London, London, UK.

[3] Athens Breath Centre, Athens, Greece.

[4] First Department of Surgery, National and Kapodistrian University of Athens, Laikon General Hospital, Athens, Greece.

[5] Department of Respiratory Medicine, Galway University Hospital, Galway, Ireland.

[6] Department of Laboratory Haematology, General Hospital for Chest Diseases "Sotiria", Athens, Greece.

[7] Division of Evolution and Genomic Science, The University of Manchester, Manchester, UK.

[8] Department of Hygiene Epidemiology and Medical Statistics, Medical School, National and Kapodistrian University of Athens, Athens, Greece.

[9] Division of Infection, Immunity and Respiratory Medicine, The University of Manchester, Manchester, UK.

[10] Allergy Department, 2nd Paediatric Clinic, National and Kapodistrian University of Athens, Athens, Greece.

[11] North West Lung Centre, Wythenshawe Hospital, Manchester University NHS Foundation Trust, Manchester Academic Health Science Centre, Manchester, UK.

[12] Institute of Infection and Global Health, University of Liverpool, Liverpool, UK.

Corresponding Author:

Alexander G. Mathioudakis MD, MRCP(UK),
Clinical Research Fellow and Honorary Lecturer in Respiratory Medicine,
North West Lung Centre, Manchester University NHS Foundation Trust, and
Division of Infection, Immunity and Respiratory Medicine, The University of Manchester,
Manchester, UK.
E-mail: alexander.mathioudakis@manchester.ac.uk.

For peer review only

Author Contribution: JV, AB and AGM contributed to conception of the study; AMK, RF, PK, JV, AB, AGM contributed to study design; RF and AGM provided methodological input; AMK, AB and AGM drafted the manuscript; JV, AB and AGM are the guarantors of this review; all authors contributed to critical revision of the manuscript for intellectual content.

Funding - Acknowledgement: This study was not supported by external funding. JV and AGM were supported by the National Institute of Health Research Manchester Biomedical Research Centre (NIHR Manchester BRC).

Declarations of interest: All authors have completed the ICMJE uniform disclosure form. None of the authors had any conflicts of interest directly related to this work. DP reports grants form Gilead Sciences, GSK, Janssen and MSD, outside the submitted work. NGP reports personal fees from Novartis, Nutricia, HAL, Menarini/Faes Farma, Sanofi, Mylan/Meda, Biomay, AstraZeneca, GSK, MSD, Asit Biotech, Boehringer Ingelheim and grans from Gerolymatos International SA and Capricare, outside the submitted work. JV reports personal fees from Chiesi Pharmaceuticals, Boehringer Ingelheim, Novartis, AstraZeneca, GSK, outside the submitted work. AGM reports grants from Boehringer Ingelheim, outside the submitted work.

Abstract

Introduction: Both stable COPD and acute exacerbations, leading causes of death, disability and healthcare expenditure, are complex, heterogeneous and poorly understood. The role of respiratory viruses has been studied extensively but is still not adequately addressed clinically. Through a rigorous evidence update, we aim to define the prevalence and clinical burden of the different respiratory viruses in stable COPD and exacerbations and to investigate whether viral load of usual respiratory viruses could be used for diagnosis of exacerbations triggered by viruses, which are currently not diagnosed or treated aetiologically.

Methods and Analysis: Based on a prospectively registered protocol, we will systematically review the literature using standard methods recommended by the Cochrane Collaboration and the Grading of Recommendations Assessment, Development and Evaluation (GRADE) Working Group. Specifically, we will evaluate: (A) the prevalence of respiratory viruses in stable COPD and exacerbations, (B) differences in the viral loads of respiratory viruses in stable COPD versus exacerbations, to explore whether the viral load of prevalent respiratory viruses could be used as a diagnostic biomarker for exacerbations triggered by viruses, (C) the association between the presence of respiratory viruses and clinical outcomes in stable COPD and in exacerbations.

Ethics and Dissemination: Ethics approval is not required since no primary data will be collected. Our findings will be presented in national and international scientific conferences and will be published in peer reviewed journals. Respiratory viruses currently represent a lost opportunity to

For peer review only

improve the outcomes of both stable COPD and exacerbations. Our work aspires to "demystify" the prevalence and clinical burden of viruses in stable COPD and exacerbations and to promote clinical and translational research.

Systematic Review Registration: This systematic review was prospectively registered with the International Prospective Register of Systematic Reviews (PROSPERO ID: CRD42019147658)

Keywords: COPD, Exacerbations, Respiratory Viruses, Prevalence, Clinical Outcomes, Biomarkers, Systematic Review, Meta-analysis, Study protocol.

Strengths and limitations of this study

- A holistic approach to a clinically pertinent question that could pump-prime clinical and translational research and facilitate the introduction of precision medicine interventions both for stable COPD and exacerbations.
- A rigorous methodology that includes a thorough evaluation of the literature, appropriate evaluation of the risk of bias of individual studies and the quality of the body of evidence, and quantitative synthesis.
- Several planned subgroup analyses to account for potential confounding; however, a potential limitation is data may not be available for the completion of all subgroup analyses.
- A prospectively published protocol increases the transparency and allowed for peer-review of the methodology utilized.

For peer review only

Introduction

Chronic obstructive pulmonary disease (COPD), a leading cause of death, disability and health related expenditure globally, is characterised by chronic debilitating respiratory symptoms and acute exacerbations, that drive the adverse outcomes of the disease[1-3]. Both stable COPD and exacerbations are complex and heterogenous, meaning that several distinct mechanistic pathways contribute to their development and progression[4]. While some of these pathways are well-established, our understanding of the pivotal role that respiratory viruses appear to have both in stable disease and exacerbations, is still lacking.

Viruses are identified in over 10% of all patients with stable COPD at any given time[5,6] and in 30-50% of those experiencing exacerbations[7-9]. In both cases, the presence of viruses is associated with worse clinical outcomes[5,6]. For example, the prevalence and clinical implications of viruses were tested longitudinally in a subgroup of 127 patients with COPD, who experienced 355 exacerbations during one year of follow-up from the AERIS cohort[6] and 83 patients, who experienced 168 exacerbations during follow-up, from the East London COPD cohort[5]. In the first cohort, viruses were present in 13.6% and 41.3% of samples received during stable disease and exacerbations, respectively. Almost half of the participants (46.5%) had at least one exacerbation that tested positive for human rhinovirus (HRV) during one year of follow-up. In the East London COPD cohort, viruses were detected in 39.2% of all exacerbations and were associated with higher symptoms burden and delayed recovery. The presence of viruses during stable state was associated with more frequent exacerbations[5]. In general, rhinovirus, influenza and respiratory syncytial virus are the most frequently detected and their clinical burden has been demonstrated in several studies[5-7,9].

Diagnostic techniques for exacerbations triggered by respiratory viruses are currently inadequate. Respiratory viruses are prevalent both in exacerbations and stable COPD. Therefore, the presence of viruses in the airways cannot confirm viral aetiology of an exacerbation, since viruses may represent incidental findings, rather than the real cause of the exacerbation.

Acute respiratory viral infections in healthy people are characterised by quick viral replication leading to high viral shedding and load, that peak rapidly[10]. Assuming that the same pattern is followed in patients with COPD, we would anticipate higher viral loads in exacerbations triggered by viruses (compared to stable COPD, but also compared to exacerbations in which viruses are present but not the causative factor). Indeed, previous studies have revealed 10-fold higher mean viral loads of human rhinovirus in exacerbations characterized by symptoms consistent with a respiratory viral infection, compared to stable COPD[8,11], suggesting that viral load might be used as a diagnostic biomarker for exacerbations triggered by viruses. Accurate diagnosis of acute exacerbations triggered by viruses could lead to the introduction of targeted antiviral treatments that are already commercially available, or in development[12]. Additionally, it could lead to a reduction of the unnecessary administration of antibiotics for exacerbations that are triggered by viruses (and not by bacteria)[13,14].

For peer review only

There is an astonishing imbalance between the ample published evidence on the prevalence and clinical impact of respiratory viruses in COPD and the lack of insight on their clinical approach and management. To improve our understanding and facilitate the introduction of precision medicine interventions for this group of patients, we will conduct a systematic review aiming to holistically evaluate:

A. The prevalence of respiratory viruses in patients with stable COPD and exacerbations.
B. Differences in the viral loads of respiratory viruses in stable COPD versus exacerbations, to explore whether viral loads of prevalent respiratory viruses could be used as a gold standard for the diagnosis of exacerbations triggered by those viruses.
C. The association between the presence of respiratory viruses and clinical outcomes of patients with stable COPD and exacerbations.

Methods and Analysis

This systematic review and meta-analysis will be based on a prospectively registered protocol at the International Prospective Register of Systematic Reviews (PROSPERO ID: CRD42019147658) and it will be conducted following standard methodology recommended by the Cochrane Collaboration[15] and the Grading of Recommendations Assessment, Development, and Evaluation (GRADE) Working Group[16]. The Preferred Reporting Items for Systematic Reviews and Meta-analyses (PRISMA) statement was used for reporting this protocol (PRISMA-p) and will also be followed for the preparation of the final report of this systematic review[17].

Eligibility criteria

We will include studies evaluating patients aged over 40 years with COPD, either during stable disease state or exacerbations. We will consider eligible studies evaluating stable COPD, provided that participants had a clinical diagnosis of COPD, confirmed by spirometry. For studies evaluating patients during exacerbations, a previous clinical diagnosis of COPD will suffice. Spirometry during an exacerbation has poor specificity, and for this reason, it will not be a prerequisite for studies on exacerbations[18,19]. We will exclude studies focusing on specific populations who are at high risk of viral infections, such as lung transplant recipients, or people with immune deficiency.

Only studies using molecular techniques to identify respiratory viruses will be included. For projects A and B (evaluating the prevalence and loads of respiratory viruses), we will include studies exploring the prevalence of respiratory viruses in representative samples of patients with stable COPD or exacerbations. Only studies assessing associations between the presence and/or load of respiratory viruses and clinical outcomes of COPD or exacerbations will be included in project C.

We will include studies of any design that could contribute data pertinent to the review questions. Data from randomized controlled trials (RCTs) will be used more cautiously compared to observational studies. More specifically, we will only include data that is unlikely to be affected by

For peer review only

investigational medications that are not part of the usual care for COPD or exacerbations. For example, in a trial evaluating the impact of a novel immunomodulatory agent versus usual care on exacerbations triggered by viruses, we will only capture data on the exacerbations' outcomes from the control group. We would capture data on the prevalence and loads of respiratory viruses from both study groups, provided they were measured prior to the initiation of antiviral treatments, in the intervention group.

Outcome Measures

Projects A, B: Primary outcome: Prevalence of respiratory viruses in the respiratory tract of patients with stable COPD and exacerbations. We will assess individual viruses separately, but also the presence of any virus. Secondary outcomes: Mean viral loads of respiratory viruses during stable COPD and exacerbations; seasonal variability in the prevalence and viral loads of respiratory viruses; proportion of patients testing positive for more than one virus. We will also describe the molecular techniques and laboratory tests used for identification and quantification of respiratory viruses.

For studies evaluating stable COPD, the main analysis will only include the first assessment of respiratory viruses in every patient. In case of multiple measurements during different seasons of the year, we will capture the first measurement during every season, to be included in the analysis of seasonal variability. For studies evaluating exacerbations, the main analysis will include the first assessment of respiratory viruses during every exacerbation. The unit of analysis will be the exacerbation, rather than the patient. In a sensitivity analysis, we will only include the first exacerbation captured for every participant.

Project C: Co-primary outcomes for studies evaluating stable COPD: Annual rate of moderate or severe exacerbations; mortality. Secondary outcomes: Annual rate of severe exacerbations; symptoms severity; health related quality of life; exercise capacity; forced expiratory volume in 1 second (FEV_1) decline rate.

Co-primary outcomes for studies evaluating exacerbations: Mortality; treatment failure rate. Secondary outcomes: treatment success rate; symptoms severity; symptoms duration; length of hospitalization; time-to-next exacerbation; exacerbations frequency; co-existing bacterial infection; proportion of patients who received antiviral treatment.

Treatment failure in exacerbations is usually defined as a composite outcome including several of the following components: lack of clinical improvement, symptoms deterioration, hospital admission, intensive care unit admission, need for additional treatments, or death[20]. Treatment success is defined as complete resolution or significant improvement of the symptoms[20].

All clinical outcomes will primarily be evaluated at longest follow-up, with the exception of treatment failure and treatments success rates in studies evaluating exacerbations, that will be evaluated at 1-4 weeks from presentation. In additional analyses of stable COPD studies, we will evaluate

For peer review only

separately data collected between 0 to <3, 3 to <6, 6 to <12 and ≥12 months of follow-up. For exacerbations studies, in additional analyses we will assess separately data collected between 0 to <1, 1 to <2, 2 to <6 or ≥6 months of follow-up, to better capture the early outcomes of exacerbations.

Literature searches

Using appropriate controlled vocabulary and free search terms, we have developed comprehensive search strategies to identify relevant published work in the electronic databases of Medline/Pubmed, EMBASE and the Cochrane Central. In addition, we will search for relevant studies in the conference proceedings of the European Respiratory Society, American Thoracic Society and Asian Pacific Society of Respirology, European Society of Clinical Microbiology and Infectious Diseases, American Society of Microbiology, European Society for Clinical Virology, in the World Health Organization International Clinical Registry Platform (ICTRP) and in the reference lists of all included studies and all previously published systematic reviews. Detailed search strategies are available in the online appendix.

Two investigators will independently screen the titles and abstracts of all studies that our searches will yield. Next, the full-text versions of all potentially eligible manuscripts and abstracts will be acquired and reviewed for confirmation of eligibility by two authors, independently. Disagreement in this and all following steps of the systematic review process will be resolved by discussion or adjudication by a third investigator, when necessary.

Data abstraction

The full reference of each eligible study, as well as details on the study design, eligibility criteria, baseline characteristics, details on the viruses evaluated and the performance characteristics of laboratory assays used for viral identification and/or quantification will be extracted by one person and will be cross-checked by another person for accuracy. Data regarding the prevalence and viral loads of respiratory viruses and all clinical outcomes will be extracted by two investigators independently.

Risk of bias assessment

For projects A and B, which focus on the prevalence and viral loads of viruses in the respiratory tract of patients with stable COPD or exacerbations, we will use the risk of bias tool for prevalence studies developed by Hoy and colleagues[21]. For project C, we will use the Newcastle-Ottawa Scale[22]. Both tools were developed for assessing the quality of nonrandomized studies. For this reason, they assess more thoroughly the representativeness of the population of the included studies[21,22]. On the contrary, tools evaluating the risk of bias in RCTs, focus less on representativeness[15]. However, representativeness of the participants is of paramount importance in our meta-analysis, which do not focus on the trial interventions. For these reasons we decided to use risk of bias tools for nonrandomized studies for risk of bias evaluation in studies of any design, including RCTs. Risk of bias will be assessed by two investigators independently.

For peer review only

If we can pool >10 studies, we will explore possible small study and publication biases by creating and examining a funnel plot and by using Egger's regression and Begg's rank correlation[15].

Data synthesis

Heterogeneity among the studies in each meta-analysis will be estimated using the I^2 statistic. Substantial heterogeneity ($I^2 \geq 50\%$) will be reported and possible causes will be explored by prespecified subgroup analyses.

In the primary analyses, data will be synthesized using a random-effects model because we anticipate significant clinical and methodological heterogeneity among the included studies. Fixed effects models will be used in sensitivity analyses. In the main analyses, individual viruses will be evaluated separately. In an additional analysis, we will evaluate the presence of any virus. Meta-analyses will be performed using R statistical software (R Foundation for Statistical Computing, Vienna, Austria) and the CRAN packages for meta-analysis (meta and metafor).

Subgroup and sensitivity analyses

If adequate data is available, we plan to carry out the following subgroup analyses:

(a) We will assess different virus subtypes or genotypes separately, as they may have different behaviour and clinical impact.
(b) We will evaluate results according to the season of the year when data and samples were collected.
(c) We will analyse separately patients who were receiving/ not receiving inhaled corticosteroids.
(d) We will analyse separately patients with different disease severity, classified based on their forced expiratory volume in 1 second (FEV_1: ≥80%, 50-80%, 30-50% or <30%). We decided not to include exacerbations history in the evaluation of disease severity, as frequency of exacerbations is an important outcome, that could be impacted by the presence of viruses.
(e) We will analyse separately patients who were tested positive for bacteria as well (project C).
(f) We will analyse separately participants with exacerbations testing positive for viruses that received versus not received antiviral treatments (project C).

Moreover, we plan to conduct the following sensitivity analyses:

(a) For analyses focusing on stable COPD, we will only include patients who did not have any exacerbations and had not received any oral corticosteroids for at least three months prior to sampling.
(b) For analyses focusing on exacerbations, we will only include those only including patients with a clinical diagnosis of COPD confirmed by spirometry during stable state.
(c) For analyses focusing on exacerbations, for every patient we will only include the first exacerbation that was captured.
(d) We will repeat meta-analyses using fixed-effect models.
(e) We will only include studies of low risk of bias.

For peer review only

Certainty of evidence

For each outcome, we will evaluate the quality of the body of evidence using GRADE methodology, which takes into consideration study limitations, consistency of the effect, imprecision, indirectness, publication bias, magnitude of effect, dose response and confounders likely minimising the effect[16].

Protocol deviations

We will conduct our systematic review according to this published protocol. Any deviations will be documented and justified in our final report.

Patient and Public Involvement

This systematic review protocol was developed without patient or public involvement.

Ethics and Dissemination

Ethical approval is not a requirement since no primary data will be collected.

The findings of this systematic review and meta-analysis will be presented in national and international scientific conferences. They will also be submitted for publication in high-impact peer reviewed journals. It is anticipated that our findings will facilitate future research and inform clinical practice.

Discussion

Respiratory viral infections represent a major unmet treatment need in both stable COPD and exacerbations. The planned meta-analyses will systemise the existing evidence on their prevalence, clinical burden and -in the case of exacerbations- diagnostic techniques.

The prevalence of viral infections in patients with COPD exacerbations has been evaluated in two previous systematic reviews. Both had limitations. Only PubMed was searched, and the most recent literature search was conducted in March 2017. When comparing the studies included in each of these reviews, we can see that important studies have been missed. Moreover, there is no formal assessment of the risk of bias of the included studies. Finally, quantitative analyses did not evaluate the prevalence of specific viruses, but rather the presence of any virus. To our knowledge, the prevalence of viral infections in patients with stable COPD and the clinical implications of the presence of viruses in either stable COPD or exacerbations has not been previously assessed in a systematic review.

Main strengths of our SRs include the clinically pertinent review questions, thorough searches of the available literature and methodological rigour. The inclusion of several subgroup and sensitivity analyses will improve our confidence on the certainty our findings. Potential unavoidable limitations

For peer review only

include the lack of data availability on less prevalent viruses (such as metapneumovirus, which may not be assessed in many of the available studies) and technical limitations of the available studies, such as lack of standardization of the molecular techniques used to identify and quantify respiratory viruses. To address technical heterogeneity, we will report on the performance characteristics of the molecular techniques used. Challenges are anticipated in pooling viral loads, as several studies use relative quantification processes with little or no normalisation. In the main analysis, evaluating the mean viral loads during stable COPD and exacerbations, only studies reporting viral copies will be included. In an additional analysis, evaluating the relation of viral loads between stable COPD and exacerbations, we will also include studies using the same relative quantification process for both disease states.

We expect that this work could pump-prime clinical and translational research, facilitate the introduction of precision medicine interventions both for stable COPD and exacerbations and improve the outcomes of these patients.

References

1. Collaborators GBDCRD. Global, regional, and national deaths, prevalence, disability-adjusted life years, and years lived with disability for chronic obstructive pulmonary disease and asthma, 1990-2015: a systematic analysis for the Global Burden of Disease Study 2015. Lancet Respir Med 2017;5:691-706.
2. Collaborators GBDCoD. Global, regional, and national age-sex specific mortality for 264 causes of death, 1980-2016: a systematic analysis for the Global Burden of Disease Study 2016. Lancet 2017;390:1151-210.
3. Vogelmeier CF, Criner GJ, Martinez FJ, et al. Global Strategy for the Diagnosis, Management, and Prevention of Chronic Obstructive Lung Disease 2017 Report: GOLD Executive Summary. Eur Respir J 2017;49.
4. Agusti A, Bel E, Thomas M, et al. Treatable traits: toward precision medicine of chronic airway diseases. Eur Respir J 2016;47:410-9.
5. Seemungal T, Harper-Owen R, Bhowmik A, et al. Respiratory viruses, symptoms, and inflammatory markers in acute exacerbations and stable chronic obstructive pulmonary disease. Am J Respir Crit Care Med 2001;164:1618-23.
6. Wilkinson TMA, Aris E, Bourne S, et al. A prospective, observational cohort study of the seasonal dynamics of airway pathogens in the aetiology of exacerbations in COPD. Thorax 2017;72:919-27.
7. Bafadhel M, McKenna S, Terry S, et al. Acute exacerbations of chronic obstructive pulmonary disease: identification of biologic clusters and their biomarkers. Am J Respir Crit Care Med 2011;184:662-71.
8. George SN, Garcha DS, Mackay AJ, et al. Human rhinovirus infection during naturally occurring COPD exacerbations. Eur Respir J 2014;44:87-96.
9. Papi A, Bellettato CM, Braccioni F, et al. Infections and airway inflammation in chronic obstructive pulmonary disease severe exacerbations. Am J Respir Crit Care Med 2006;173:1114-21.
10. Lee N, Chan PK, Hui DS, et al. Viral loads and duration of viral shedding in adult patients hospitalized with influenza. J Infect Dis 2009;200:492-500.
11. Quint JK, Donaldson GC, Goldring JJ, Baghai-Ravary R, Hurst JR, Wedzicha JA. Serum IP-10 as a biomarker of human rhinovirus infection at exacerbation of COPD. Chest 2010;137:812-22.
12. Casanova V, Sousa FH, Stevens C, Barlow PG. Antiviral therapeutic approaches for human rhinovirus infections. Future Virol 2018;13:505-18.
13. Tickoo M, Ruthazer R, Bardia A, et al. The effect of respiratory viral assay panel on antibiotic prescription patterns at discharge in adults admitted with mild to moderate acute exacerbation of COPD: a retrospective before- after study. BMC Pulm Med 2019;19:118.

For peer review only

14. Mathioudakis AG, Chatzimavridou-Grigoriadou V, Corlateanu A, Vestbo J. Procalcitonin to guide antibiotic administration in COPD exacerbations: a meta-analysis. Eur Respir Rev 2017;26(143). pii: 160073.
15. Higgins JPT. Cochrane Handbook for Systematic Reviews of Interventions. The Cochrane Collaboration. 2011.
16. Guyatt GH, Oxman AD, Vist GE, et al. GRADE: an emerging consensus on rating quality of evidence and strength of recommendations. BMJ 2008;336:924-6.
17. Moher D, Liberati A, Tetzlaff J, Altman DG, Group P. Preferred reporting items for systematic reviews and meta-analyses: the PRISMA statement. J Clin Epidemiol 2009;62:1006-12.
18. Loh CH, Genese FA, Kannan KK, Lovings TM, Peters SP, Ohar JA. Spirometry in Hospitalized Patients with Acute Exacerbation of COPD Accurately Predicts Post Discharge Airflow Obstruction. Chronic Obstr Pulm Dis 2018;5:124-33.
19. Mathioudakis AG, Janner J, Moberg M, Alonso-Coello P, Vestbo J. A systematic evaluation of the diagnostic criteria for COPD and exacerbations used in randomized controlled trials on the management of COPD exacerbations. ERJ Open Res 2019.
20. Mathioudakis AG, Moberg M, Janner J, Alonso-Coello P, Vestbo J. Outcomes reported on the management of COPD exacerbations: a systematic survey of randomised controlled trials. ERJ Open Res 2019;5(2). pii: 00072-2019.
21. Hoy D, Brooks P, Woolf A, et al. Assessing risk of bias in prevalence studies: modification of an existing tool and evidence of interrater agreement. J Clin Epidemiol 2012;65:934-9.
22. Wells GA, Shea B, O'Connell D, et al. The Newcastle-Ottawa Scale (NOS) for assessing the quality if nonrandomized studies in meta-analyses. (Accessed 18/08/2019, at: http://www.ohri.ca/programs/clinical_epidemiology/oxford.htm).

Author E-mails:

Anastasia M. Kefala: anastasia.m.kefala@gmail.com,

Rebecca Fortescue: rnormans@sgul.ac.uk,

Gioulinta S. Alimani: juli.alimani95@gmail.com,

Prodromos Kanavidis: makis.k@gmail.com,

Melissa Jane McDonnell: melissajanemcdonnell@gmail.com,

Emmanouil Magiorkinis: emmanouil.magiorkinis@gmail.com,

Spyridon Megremis: spyridon.megremis@manchester.ac.uk,

Dimitrios Paraskevis: dparask@med.uoa.gr,

Chrysa Voyiatzaki: cvoyiatz@uniwa.gr,

Georgios A. Mathioudakis: geoamath@gmail.com,

Effie Papageorgiou: efipapag@uniwa.gr,

Nikolaos G. Papadopoulos: ngpallergy@gmail.com,

Jørgen Vestbo: Jorgen.Vestbo@manchester.ac.uk,

Apostolos Beloukas: abeloukas@uniwa.gr,

Alexander G. Mathioudakis: Alexander.Mathioudakis@manchester.ac.uk.

For peer review only

Online Appendix

Title: Prevalence and clinical implications of respiratory viruses in stable chronic obstructive pulmonary disease (COPD) and exacerbations: A systematic review and meta-analysis protocol.

Authors: Anastasia M. Kefala, Rebecca Fortescue, Gioulinta S. Alimani, Prodromos Kanavidis, Melissa Jane McDonnell, Emmanouil Magiorkinis, Dimitrios Paraskevis, Chrysa Voyiatzaki, Georgios A. Mathioudakis, Nikolaos G. Papadopoulos, Jørgen Vestbo, Apostolos Beloukas, Alexander G. Mathioudakis.

Search Strategy - Medline, PubMed & Cochrane Library

#1 Chronic Obstructive Pulmonary Disease [MH]

#2 Lung Diseases, Obstructive [MH:NOEXP]

#3 Emphysema [MH]

#4 Chronic Bronchitis [MH]

#5 COPD [tiab]

#6 COAD [tiab]

#7 "Chronic Bronchitis" [tiab]

#8 Emphysema [tiab]

#9 Obstructive[ti]

#10 (Pulmonary OR Respiratory OR Airway OR Airflow OR Lung)[ti]

#11 #9 AND #10

#12 AECOPD[tiab]

#13 IECOPD[tiab]

#14 #1 OR #2 OR #3 OR #4 OR #5 OR #6 OR #7 OR #8 OR #11 OR #12 OR #13

#15 Viruses[MH]

#16 Influenza, Human[MH]

#17 Rhinovirus[MH]

#18 Respiratory Syncytial Viruses[MH]

#19 Coronavirus[MH]

#20 Paramyxoviridae Infections[MH]

#21 Orthomyxoviridae[MH]

#22 Adenoviridae [MH]

For peer review only

#23 Picornaviridae[MH]

#24 Metapneumovirus[MH]

#25 Enterovirus[MH]

#26 Cytomegalovirus[MH]

#27 Herpesvirus 3, Human[MH]

#28 Virus*[tiab]

#29 Viral*[tiab]

#30 Influenza*[tiab]

#31 Rhinovir*[tiab]

#32 Respiratory Syncytial Vir*[tiab]

#33 Coronavir*[tiab]

#34 Paramyxovir*[tiab]

#35 Orthomyxovir*[tiab]

#36 Adenovir*[tiab]

#37 Picornavir*[tiab]

#38 Metapneumov*[tiab]

#39 vzv[tiab]

#40 varicella[tiab]

#41 Enterovir*[tiab]

#42 Parainfluenza[tiab]

#43 Echovir*[tiab]

#44 #15 or #16 or #17 or #18 or #19 or #20 or #21 or #22 or #23 or #24 or #25 or #26 or #27 or #28 or #29 or #30 or #31 or #32 or #33 or #34 or #35 or #36 or #37 or #38 or #39 or #40 or #41 or #42 or #43

#45 Animals[mh] not (humans[mh])

#46 (child[mh]or (adolescent[mh])) not (adult[mh])

#47 editorial[pulication type]

#48 review[publication type] not (systematic review [publication type])

#49 #14 and #44

#50 #49 NOT (#45 or #46 or #47 or #48)

For peer review only

Search Strategy: EMBASE

1 exp Chronic Obstructive Pulmonary Disease/

2 exp Lung diseases, obstructive/

3 exp Chronic bronchitis/

4 exp emphysema/

5 COPD.tw.

6 COAD.tw.

7 (chronic adj2 bronchit$).tw.

8 (obstructive adj3 (pulmonary or lung$ or airway$ or airflow$ or bronch$ or respirat$)).tw.

9 AECOPD.tw

10 IECOPD.tw

11 ECOPD.tw

12 1 or 2 or 3 or 4 or 5 or 6 or 7 or 8 or 9 or 10 or 11

13 exp virus/

14 exp influenza/

15 exp rhinovirus/

16 exp Respiratory Syncytial Virus/

17 exp Coronavirus/

18 exp Paramyxoviridae Infections/

19 exp Orthomyxoviridae/

20 exp Adenoviridae/

21 exp Picornaviridae/

22 exp Metapneumovirus/

23 exp Enterovirus/

24 exp Cytomegalovirus/

25 exp bocavirus/

26 virus$.tw.

27 viral$.tw.

28 Influenza$.tw.

29 (("haemophilus influenzae" or "h. influenza") not influenza).tw.

For peer review only

30 28 not 29

31 Rhinovir$.tw.

32 Respiratory Syncytial Vir$.tw.

33 Coronavir$.tw.

34 Paramyxovir$.tw.

35 Orthomyxovir$.tw.

36 Adenovir$.tw.

37 Picornavir$.tw.

38 Metapneumov$.tw.

39 Enterovir$.tw.

40 Parainfluenza.tw.

41 Echovir$.tw.

42 Bocavir$.tw.

43 vzv.tw.

44 varicella.tw.

45 13 or 14 or 15 or 16 or 17 or 18 or 19 or 20 or 21 or 22 or 23 or 24 or 25 or 26 or 27 or 30 or 31 or 32 or 33 or 34 or 35 or 36 or 37 or 38 or 39 or 40 or 41 or 42 or 43 or 44

46 exp animals/ not exp humans/

47 (exp child/ or exp adolescent/) not exp adult/

48 exp editorial/ or (exp review/ not (exp systematic review/))

49 12 and 45

50 49 not (46 or 47 or 48)

For peer review only

Prevalence and clinical implications of respiratory viruses in stable chronic obstructive pulmonary disease (COPD) and exacerbations: A systematic review and meta-analysis protocol.

Anastasia M. Kefala, Rebecca Fortescue, Gioulinta S. Alimani, Prodromos Kanavidis, Melissa Jane McDonnell, Emmanouil Magiorkinis, Spyridon Megremis, Dimitrios Paraskevis, Chrysa Voyiatzaki, Georgios A. Mathioudakis, Effie Papageorgiou, Nikolaos G. Papadopoulos, Jørgen Vestbo, Apostolos Beloukas, Alexander G. Mathioudakis.

For peer review only

PRISMA-P 2015 Checklist

This checklist has been adapted for use with protocol submissions to *Systematic Reviews* from Table 3 in Moher D et al: Preferred reporting items for systematic review and meta-analysis protocols (PRISMA-P) 2015 statement. *Systematic Reviews* 2015 **4**:1

Section/topic	#	Checklist item	Information reported		Line number(s)
			Yes	No	
**ADMINISTRATIVE INFORMATION**					
**Title**					
Identification	1a	Identify the report as a protocol of a systematic review	☒	☐	1-2
Update	1b	If the protocol is for an update of a previous systematic review, identify as such	☐	☒	N/A
**Registration**	2	If registered, provide the name of the registry (e.g., PROSPERO) and registration number in the Abstract	☒	☐	79-80
**-Authors**					
Contact	3a	Provide name, institutional affiliation, and e-mail address of all protocol authors; provide physical mailing address of corresponding author	☒	☐	4-37, 379-394
Contributions	3b	Describe contributions of protocol authors and identify the guarantor of the review	☒	☐	41-44
**Amendments**	4	If the protocol represents an amendment of a previously completed or published protocol, identify as such and list changes; otherwise, state plan for documenting important protocol amendments	☒	☐	280-282
**Support**					
Sources	5a	Indicate sources of financial or other support for the review	☒	☐	45-47
Sponsor	5b	Provide name for the review funder and/or sponsor	☐	☒	N/A

Section/topic	#	Checklist item	Information reported Yes	Information reported No	Line number(s)
Role of sponsor/funder	5c	Describe roles of funder(s), sponsor(s), and/or institution(s), if any, in developing the protocol	☐	☒	N/A
**INTRODUCTION**					
**Rationale**	6	Describe the rationale for the review in the context of what is already known	☒	☐	95-131
**Objectives**	7	Provide an explicit statement of the question(s) the review will address with reference to participants, interventions, comparators, and outcomes (PICO)	☒	☐	132-1143, Methods
**METHODS**					
**Eligibility criteria**	8	Specify the study characteristics (e.g., PICO, study design, setting, time frame) and report characteristics (e.g., years considered, language, publication status) to be used as criteria for eligibility for the review	☒	☐	152-173
**Information sources**	9	Describe all intended information sources (e.g., electronic databases, contact with study authors, trial registers, or other grey literature sources) with planned dates of coverage	☒	☐	207-216
**Search strategy**	10	Present draft of search strategy to be used for at least one electronic database, including planned limits, such that it could be repeated	☒	☐	207-216, online appendix
STUDY RECORDS					
Data management	11a	Describe the mechanism(s) that will be used to manage records and data throughout the review	☒	☐	217-221
Selection process	11b	State the process that will be used for selecting studies (e.g., two independent reviewers) through each phase of the review (i.e., screening, eligibility, and inclusion in meta-analysis)	☒	☐	217-221
Data collection process	11c	Describe planned method of extracting data from reports (e.g., piloting forms, done independently, in duplicate), any processes for obtaining and confirming data from investigators	☒	☐	222-228
**Data items**	12	List and define all variables for which data will be sought (e.g., PICO items, funding sources), any pre-planned data assumptions and simplifications	☒	☐	222-228
**Outcomes and prioritization**	13	List and define all outcomes for which data will be sought, including prioritization of main and additional outcomes, with rationale	☒	☐	174-206
**Risk of bias in individual studies**	14	Describe anticipated methods for assessing risk of bias of individual studies, including whether this will be done at the outcome or study level, or both; state how this information will be used in data synthesis	☒	☐	229-241
DATA					
**Synthesis**	15a	Describe criteria under which study data will be quantitatively synthesized	☒	☐	242-251
	15b	If data are appropriate for quantitative synthesis, describe planned summary measures, methods of handling data, and methods of combining data from studies, including any planned exploration of consistency (e.g., I^2, Kendall's tau)	☒	☐	243-245

For peer review only

Section/topic	#	Checklist item	Information reported		Line number(s)
			Yes	No	
	15c	Describe any proposed additional analyses (e.g., sensitivity or subgroup analyses, meta-regression)	☒	☐	252-275
	15d	If quantitative synthesis is not appropriate, describe the type of summary planned	☐	☒	N/A
**Meta-bias(es)**	16	Specify any planned assessment of meta-bias(es) (e.g., publication bias across studies, selective reporting within studies)	☒	☐	240-241
**Confidence in cumulative evidence**	17	Describe how the strength of the body of evidence will be assessed (e.g., GRADE)	☒	☐	276-279

For peer review only

BMJ Open

Prevalence and clinical implications of respiratory viruses in stable chronic obstructive pulmonary disease (COPD) and exacerbations: A systematic review and meta-analysis protocol.

Journal:	*BMJ Open*
Manuscript ID	bmjopen-2019-035640.R1
Article Type:	Protocol
Date Submitted by the Author:	20-Jan-2020
Complete List of Authors:	Kefala, Anastasia; University of West Attica, Department of Biomedical Sciences<br>Fortescue, Rebecca ; University of London Saint George's, Cochrane Airways, Population Health Research Institute<br>Alimani, Gioulinta; Athens Breath Centre; University of West Attica, Department of Biomedical Sciences<br>Kanavidis, Prodromos; National and Kapodistrian University of Athens, First Department of Surgery<br>McDonnell, Melissa Jane; Galway University Hospitals, Department of Respiratory Medicine<br>Magiorkinis, Emmanouil; University of West Attica, Department of Biomedical Sciences; Sotiria Regional Chest Disease Hospital of Athens, Department of Laboratory Haematology<br>Megremis, Spyridon; The University of Manchester, Division of Evolution and Genomic Science<br>Paraskevis, Dimitrios; National and Kapodistrian University of Athens, Department of Hygiene Epidemiology and Medical Statistics<br>Voyiatzaki, Chrysa; University of West Attica, Department of Biomedical Sciences<br>Mathioudakis, Georgios; Athens Breath Centre<br>Papageorgiou, Effie; University of West Attica, Department of Biomedical Sciences<br>Papadopoulos, Nikolaos; The University of Manchester, Division of Infection, Immunity and Respiratory Medicine; National and Kapodistrian University of Athens, Allergy Department, 2nd Paediatric Clinic<br>Vestbo, Joergen; The University of Manchester, Division of Infection, Immunity and Respiratory Medicine; Manchester University NHS Foundation Trust, North West Lung Centre<br>Beloukas, Apostolos; University of West Attica, Department of Biomedical Sciences<br>Mathioudakis, Alexander; The University of Manchester, Division of Infection, Immunity and Respiratory Medicine; Manchester University NHS Foundation Trust, North West Lung Centre
**Primary Subject Heading**:	Respiratory medicine
Secondary Subject Heading:	Research methods, Evidence based practice, Epidemiology

Keywords:	Epidemiology < INFECTIOUS DISEASES, Epidemiology < THORACIC MEDICINE, Chronic airways disease < THORACIC MEDICINE, Adult thoracic medicine < THORACIC MEDICINE, Respiratory infections < THORACIC MEDICINE

SCHOLARONE™
Manuscripts

I, the Submitting Author has the right to grant and does grant on behalf of all authors of the Work (as defined in the below author licence), an exclusive licence and/or a non-exclusive licence for contributions from authors who are: i) UK Crown employees; ii) where BMJ has agreed a CC-BY licence shall apply, and/or iii) in accordance with the terms applicable for US Federal Government officers or employees acting as part of their official duties; on a worldwide, perpetual, irrevocable, royalty-free basis to BMJ Publishing Group Ltd ("BMJ") its licensees and where the relevant Journal is co-owned by BMJ to the co-owners of the Journal, to publish the Work in this journal and any other BMJ products and to exploit all rights, as set out in our licence.

The Submitting Author accepts and understands that any supply made under these terms is made by BMJ to the Submitting Author unless you are acting as an employee on behalf of your employer or a postgraduate student of an affiliated institution which is paying any applicable article publishing charge ("APC") for Open Access articles. Where the Submitting Author wishes to make the Work available on an Open Access basis (and intends to pay the relevant APC), the terms of reuse of such Open Access shall be governed by a Creative Commons licence – details of these licences and which Creative Commons licence will apply to this Work are set out in our licence referred to above.

Other than as permitted in any relevant BMJ Author's Self Archiving Policies, I confirm this Work has not been accepted for publication elsewhere, is not being considered for publication elsewhere and does not duplicate material already published. I confirm all authors consent to publication of this Work and authorise the granting of this licence.

For peer review only

Title: Prevalence and clinical implications of respiratory viruses in stable chronic obstructive pulmonary disease (COPD) and exacerbations: A systematic review and meta-analysis protocol.

Authors: Anastasia M. Kefala[1], Rebecca Fortescue[2], Gioulinta S. Alimani[1,3], Prodromos Kanavidis[4], Melissa Jane McDonnell[5], Emmanouil Magiorkinis[1,6], Spyridon Megremis[7], Dimitrios Paraskevis[8], Chrysa Voyiatzaki[1], Georgios A. Mathioudakis[3], Effie Papageorgiou[1], Nikolaos G. Papadopoulos[9,10], Joergen Vestbo[9,11], Apostolos Beloukas[1,12#], Alexander G. Mathioudakis[9,11#].

[#] AB and AGM equally shared last authorship to this work.

Affiliations:

[1] Department of Biomedical Sciences, University of West Attica, Athens, Greece.

[2] Cochrane Airways, Population Health Research Institute, St George's, University of London, London, UK.

[3] Athens Breath Centre, Athens, Greece.

[4] First Department of Surgery, National and Kapodistrian University of Athens, Laikon General Hospital, Athens, Greece.

[5] Department of Respiratory Medicine, Galway University Hospital, Galway, Ireland.

[6] Department of Laboratory Haematology, General Hospital for Chest Diseases "Sotiria", Athens, Greece.

[7] Division of Evolution and Genomic Science, The University of Manchester, Manchester, UK.

[8] Department of Hygiene Epidemiology and Medical Statistics, Medical School, National and Kapodistrian University of Athens, Athens, Greece.

[9] Division of Infection, Immunity and Respiratory Medicine, The University of Manchester, Manchester, UK.

[10] Allergy Department, 2nd Paediatric Clinic, National and Kapodistrian University of Athens, Athens, Greece.

[11] North West Lung Centre, Wythenshawe Hospital, Manchester University NHS Foundation Trust, Manchester Academic Health Science Centre, Manchester, UK.

[12] Institute of Infection and Global Health, University of Liverpool, Liverpool, UK.

Corresponding Author:

Alexander G. Mathioudakis MD, MRCP(UK),
Clinical Research Fellow and Honorary Lecturer in Respiratory Medicine,
North West Lung Centre, Manchester University NHS Foundation Trust, and
Division of Infection, Immunity and Respiratory Medicine, The University of Manchester,
Manchester, UK.
E-mail: alexander.mathioudakis@manchester.ac.uk.

For peer review only

Author Contribution: JV, AB and AGM contributed to conception of the study; AMK, RF, PK, JV, AB, AGM contributed to study design; RF and AGM provided methodological input; AMK, AB and AGM drafted the manuscript; JV, AB and AGM are the guarantors of this review; all authors (AMK, RF, GSA, PK, MJM, EM, SM, DP, CV, GAM, EP, NGP, JV, AB, AGM) contributed to critical revision of the manuscript for intellectual content.

Funding - Acknowledgement: This study was not supported by external funding. JV and AGM were supported by the National Institute of Health Research Manchester Biomedical Research Centre (NIHR Manchester BRC).

Declarations of interest: All authors have completed the ICMJE uniform disclosure form. None of the authors had any conflicts of interest directly related to this work. DP reports grants form Gilead Sciences, GSK, Janssen and MSD, outside the submitted work. NGP reports personal fees from Novartis, Nutricia, HAL, Menarini/Faes Farma, Sanofi, Mylan/Meda, Biomay, AstraZeneca, GSK, MSD, Asit Biotech, Boehringer Ingelheim and grans from Gerolymatos International SA and Capricare, outside the submitted work. JV reports personal fees from Chiesi Pharmaceuticals, Boehringer Ingelheim, Novartis, AstraZeneca, GSK, outside the submitted work. AGM reports grants from Boehringer Ingelheim, outside the submitted work.

Abstract

Introduction: Both stable COPD and acute exacerbations represent leading causes of death, disability and healthcare expenditure. They are complex, heterogeneous and their mechanisms are poorly understood. The role of respiratory viruses has been studied extensively but is still not adequately addressed clinically. Through a rigorous evidence update, we aim to define the prevalence and clinical burden of the different respiratory viruses in stable COPD and exacerbations and to investigate whether viral load of usual respiratory viruses could be used for diagnosis of exacerbations triggered by viruses, which are currently not diagnosed or treated aetiologically.

Methods and Analysis: Based on a prospectively registered protocol, we will systematically review the literature using standard methods recommended by the Cochrane Collaboration and the Grading of Recommendations Assessment, Development and Evaluation (GRADE) Working Group. We will search Medline/PubMed, EMBASE, the Cochrane Library, the World Health Organization's Clinical Trials Registry and the proceedings of relevant international conferences on March 2nd, 2020. We will evaluate: (A) the prevalence of respiratory viruses in stable COPD and exacerbations, (B) differences in the viral loads of respiratory viruses in stable COPD versus exacerbations, to explore whether the viral load of prevalent respiratory viruses could be used as a diagnostic biomarker for exacerbations triggered by viruses, (C) the association between the presence of respiratory viruses and clinical outcomes in stable COPD and in exacerbations.

For peer review only

Ethics and Dissemination: Ethics approval is not required since no primary data will be collected. Our findings will be presented in national and international scientific conferences and will be published in peer reviewed journals. Respiratory viruses currently represent a lost opportunity to improve the outcomes of both stable COPD and exacerbations. Our work aspires to "demystify" the prevalence and clinical burden of viruses in stable COPD and exacerbations and to promote clinical and translational research.

Systematic Review Registration: CRD42019147658 (PROSPERO Registry).

Keywords: COPD, Exacerbations, Respiratory Viruses, Prevalence, Clinical Outcomes, Biomarkers, Systematic Review, Meta-analysis, Study protocol.

Strengths and limitations of this study

- A holistic approach to a clinically pertinent question that could pump-prime clinical and translational research and facilitate the introduction of precision medicine interventions both for stable COPD and exacerbations.
- A rigorous methodology that includes a thorough evaluation of the literature, appropriate evaluation of the risk of bias of individual studies and the quality of the body of evidence, and quantitative synthesis.
- Several planned meta-regression analyses to explore potential effect modifying factors; however, a potential limitation is data may not be available for the completion of all these analyses.
- A prospectively published protocol increases the transparency and allowed for peer-review of the methodology utilized.

For peer review only

Introduction

Chronic obstructive pulmonary disease (COPD), a leading cause of death, disability and health related expenditure globally, is characterised by chronic debilitating respiratory symptoms and acute exacerbations, that drive the adverse outcomes of the disease[1-4]. Both stable COPD and exacerbations are complex and heterogenous, meaning that several distinct mechanistic pathways contribute to their development and progression[5]. While some of these pathways are well-established, our understanding of the pivotal role that respiratory viruses appear to have both in stable disease and exacerbations, is still lacking.

Viruses are identified in over 10% of all patients with stable COPD at any given time[6,7] and in 30-50% of those experiencing exacerbations[8-10]. In both cases, the presence of viruses is associated with worse clinical outcomes[6,7]. For example, the prevalence and clinical implications of viruses were tested longitudinally in a subgroup of 127 patients with COPD, who experienced 355 exacerbations during one year of follow-up from the AERIS cohort[7] and 83 patients, who experienced 168 exacerbations during follow-up, from the East London COPD cohort[6]. In the first cohort, viruses were present in 13.6% and 41.3% of samples received during stable disease and exacerbations, respectively. Almost half of the participants (46.5%) had at least one exacerbation that tested positive for human rhinovirus (HRV) during one year of follow-up. In the East London COPD cohort, viruses were detected in 39.2% of all exacerbations and were associated with higher symptoms burden and delayed recovery. The presence of viruses during stable state was associated with more frequent exacerbations[6]. In general, rhinovirus, influenza and respiratory syncytial virus are the most frequently detected and their clinical burden has been demonstrated in several studies[6-8,10].

Diagnostic techniques for exacerbations triggered by respiratory viruses are currently inadequate. Respiratory viruses are prevalent both in exacerbations and stable COPD. Therefore, the presence of viruses in the airways cannot confirm viral aetiology of an exacerbation, since viruses may represent incidental findings, rather than the real cause of the exacerbation.

Acute respiratory viral infections in healthy people are characterised by quick viral replication leading to high viral shedding and load, that peak rapidly[11]. Assuming that the same pattern is followed in patients with COPD, we would anticipate higher viral loads in exacerbations triggered by viruses (compared to stable COPD, but also compared to exacerbations in which viruses are present but not the causative factor). Indeed, previous studies have revealed 10-fold higher mean viral loads of human rhinovirus in exacerbations characterized by symptoms consistent with a respiratory viral infection, compared to stable COPD[9,12], suggesting that viral load might be used as a diagnostic biomarker for exacerbations triggered by viruses. Accurate diagnosis of acute exacerbations triggered by viruses could lead to the introduction of targeted antiviral treatments that are already commercially available, or in development[13]. Additionally, it could lead to a reduction of the unnecessary administration of antibiotics for exacerbations that are triggered by viruses (and not by bacteria)[14,15].

For peer review only

There is an astonishing imbalance between the ample published evidence on the prevalence and clinical impact of respiratory viruses in COPD and the lack of insight on their clinical approach and management. To improve our understanding and facilitate the introduction of precision medicine interventions for this group of patients, we will conduct a systematic review aiming to holistically evaluate:

A. The prevalence of respiratory viruses in patients with stable COPD and exacerbations.
B. Differences in the viral loads of respiratory viruses in stable COPD versus exacerbations, to explore whether viral loads of prevalent respiratory viruses could be used as a gold standard for the diagnosis of exacerbations triggered by those viruses.
C. The association between the presence of respiratory viruses and clinical outcomes of patients with stable COPD and exacerbations.

Methods and Analysis

This systematic review and meta-analysis will be based on a prospectively registered protocol at the International Prospective Register of Systematic Reviews (PROSPERO ID: CRD42019147658) and it will be conducted following standard methodology recommended by the Cochrane Collaboration[16] and the Grading of Recommendations Assessment, Development, and Evaluation (GRADE) Working Group[17]. The Preferred Reporting Items for Systematic Reviews and Meta-analyses (PRISMA) statement was used for reporting this protocol (PRISMA-P)[18] and will also be followed for the preparation of the final report of this systematic review[19].

Eligibility criteria

We will include studies evaluating patients aged over 40 years with COPD, either during stable disease state or exacerbations. We will consider eligible studies evaluating stable COPD, provided that participants had a clinical diagnosis of COPD, confirmed by spirometry. For studies evaluating patients during exacerbations, a previous clinical diagnosis of COPD will suffice. Spirometry during an exacerbation has poor specificity, and for this reason, it will not be a prerequisite for studies on exacerbations[20,21]. We will exclude studies focusing on specific populations who are at high risk of viral infections, such as lung transplant recipients, or people with immune deficiency.

Only studies using molecular techniques to identify respiratory viruses will be included. For projects A and B (evaluating the prevalence and loads of respiratory viruses), we will include studies exploring the prevalence of respiratory viruses in representative samples of patients with stable COPD or exacerbations. Only studies assessing associations between the presence and/or load of respiratory viruses and clinical outcomes of COPD or exacerbations will be included in project C.

We will include studies of any design that could contribute data pertinent to the review questions. Data from randomized controlled trials (RCTs) will be used more cautiously compared to observational studies. More specifically, we will only include data that is unlikely to be affected by

For peer review only

investigational medications that are not part of the usual care for COPD or exacerbations. For example, in a trial evaluating the impact of a novel immunomodulatory agent versus usual care on exacerbations triggered by viruses, we will only capture data on the exacerbations' outcomes from the control group. We would capture data on the prevalence and loads of respiratory viruses from both study groups, provided they were measured prior to the initiation of antiviral treatments, in the intervention group.

Outcome Measures

Projects A, B: Primary outcome: Prevalence of respiratory viruses in the respiratory tract of patients with stable COPD and exacerbations. We will assess individual viruses separately, but also the presence of any virus. Secondary outcomes: Mean viral loads of respiratory viruses during stable COPD and exacerbations; seasonal variability in the prevalence and viral loads of respiratory viruses; proportion of patients testing positive for more than one virus. We will also describe the molecular techniques and laboratory tests used for identification and quantification of respiratory viruses.

For studies evaluating stable COPD, the main analysis will only include the first assessment of respiratory viruses in every patient. In case of multiple measurements during different seasons of the year, we will capture the first measurement during every season, to be included in the analysis of seasonal variability. For studies evaluating exacerbations, the main analysis will include the first assessment of respiratory viruses during every exacerbation. The unit of analysis will be the exacerbation, rather than the patient. In a sensitivity analysis, we will only include the first exacerbation captured for every participant.

Project C: Co-primary outcomes for studies evaluating stable COPD: Annual rate of moderate or severe exacerbations; mortality. Secondary outcomes: Annual rate of severe exacerbations; symptoms severity; health related quality of life; exercise capacity; forced expiratory volume in 1 second (FEV_1) decline rate.

Co-primary outcomes for studies evaluating exacerbations: Mortality; treatment failure rate. Secondary outcomes: treatment success rate; symptoms severity; symptoms duration; length of hospitalization; time-to-next exacerbation; exacerbations frequency; co-existing bacterial infection; proportion of patients who received antiviral treatment.

Treatment failure in exacerbations is usually defined as a composite outcome including several of the following components: lack of clinical improvement, symptoms deterioration, hospital admission, intensive care unit admission, need for additional treatments, or death[22]. Treatment success is defined as complete resolution or significant improvement of the symptoms[22].

All clinical outcomes will primarily be evaluated at longest follow-up, with the exception of treatment failure and treatments success rates in studies evaluating exacerbations, that will be evaluated at 1-4 weeks from presentation. In additional analyses of stable COPD studies, we will evaluate

For peer review only

separately data collected between 0 to <3, 3 to <6, 6 to <12 and ≥12 months of follow-up. For exacerbations studies, in additional analyses we will assess separately data collected between 0 to <1, 1 to <2, 2 to <6 or ≥6 months of follow-up, to better capture the early outcomes of exacerbations.

Systematic literature search

The electronic databases of Medline/Pubmed, EMBASE and the Cochrane Central will be searched on March 2nd, 2020, using a comprehensive search strategy using appropriate controlled vocabulary and free search terms. This strategy was developed by one author (AGM) and was updated following input from all authors and identification of MeSH terms from several eligible studies that were identified during pilot searches. In addition to the online databases, we will search for relevant studies in the conference proceedings of the European Respiratory Society, American Thoracic Society and Asian Pacific Society of Respirology, European Society of Clinical Microbiology and Infectious Diseases, American Society of Microbiology, European Society for Clinical Virology, in the World Health Organization International Clinical Registry Platform (ICTRP) and in the reference lists of all included studies and all previously published systematic reviews. Detailed search strategies are available in the online appendix.

Two investigators will independently screen the titles and abstracts of all studies that our searches will yield. Next, the full-text versions of all potentially eligible manuscripts and abstracts will be acquired and reviewed for confirmation of eligibility by two authors, independently. Disagreement in this and all following steps of the systematic review process will be resolved by discussion or adjudication by a third investigator, when necessary.

Data abstraction

Relevant data from each eligible study will be extracted in a predefined, pilot-tested excel spreadsheet. The full reference of each eligible study, as well as details on the study design, eligibility criteria, baseline characteristics, details on the viruses evaluated and the performance characteristics of laboratory assays used for viral identification and/or quantification will be extracted by one person and will be cross-checked by another person for accuracy. Data regarding the prevalence and viral loads of respiratory viruses and all clinical outcomes will be extracted by two investigators independently. Details on all data and variables that will be extracted are available in the online appendix.

Pilot testing of the data extraction form on a sample of studies will be conducted by all investigators who will subsequently use the form. In case of discrepancies, additional eligible studies will be extracted until achieving an agreement in >95% of the extracted variables among all reviewers. Feedback will be sought during this process and variables may be added or modified. Missing data will be requested directly from the study investigators, via e-mail.

Risk of bias assessment

For peer review only

For projects A and B, which focus on the prevalence and viral loads of viruses in the respiratory tract of patients with stable COPD or exacerbations, we will use the risk of bias tool for prevalence studies developed by Hoy and colleagues[23]. For project C, we will use the Newcastle-Ottawa Scale[24]. Both tools were developed for assessing the quality of nonrandomized studies. For this reason, they assess more thoroughly the representativeness of the population of the included studies[23,24]. On the contrary, tools evaluating the risk of bias in RCTs, focus less on representativeness[16]. However, representativeness of the participants is of paramount importance in our meta-analysis, which does not focus on the trial interventions. For these reasons we decided to use risk of bias tools for nonrandomized studies for risk of bias evaluation in studies of any design, including RCTs. Risk of bias will be assessed by two investigators independently. For each study, we will report an overall risk of bias assessment, but also our judgement of the risk of bias related to each of the domains proposed by the selected tools.If we can pool >10 studies, we will explore possible small study and publication biases by creating and examining a funnel plot and by using Egger's regression and Begg's rank correlation[16].

Data synthesis

Heterogeneity among the studies in each meta-analysis will be estimated using the I^2 statistic. Substantial heterogeneity ($I^2 \geq 50\%$) will be reported and possible causes will be explored by prespecified meta-regression analyses.

In the primary analyses, data will be synthesized using a random-effects model because we anticipate significant clinical and methodological heterogeneity among the included studies. Fixed effects models will be used in sensitivity analyses. The inverse variance method using logit transformed proportions will be used for conducting meta-analysis of proportions. In the main analyses, individual viruses will be evaluated separately. In an additional analysis, we will evaluate the presence of any virus. Meta-analyses will be performed using R statistical software (R Foundation for Statistical Computing, Vienna, Austria) and the CRAN packages for meta-analysis (meta and metafor).

Meta-regression and sensitivity analyses

If adequate data is available, we will conduct meta-regression analyses to evaluate the potential impact of the following parameters on the outcomes: (i) the season of the year when samples were collected; (ii) use of inhaled corticosteroids; (iii) spirometric severity of COPD, classified based on their forced expiratory volume in 1 second (FEV_1: ≥80%, 50-80%, 30-50% or <30%); (iv) year of study completion; (v) study design [observational versus interventional]. Additional parameters to be tested in project C will include: (vi) bacterial co-infection; (vii) receipt of antiviral treatment. For each of these analyses, we will first attempt to use subgroup data from the included studies, if they are available to us. Alternatively, we will use the following study-level variables for each of the analyses: (i) the proportion of samples that were collected during the flu season; (ii) the proportion of patients that were using inhaled corticosteroids; (iii) the mean FEV_1 of the participants; (iv-v) same as above;

For peer review only

(vi) proportion of patients with bacterial co-infection; (vii) proportion of patients that received antiviral treatment. For conducting these analyses, we will use a random-effects model and for meta-regression analyses of proportions, we will use the inverse variance method with logit transformed proportions. We will report the level of significance of the regressions (p-value) and the correlation coefficients. A risk of type I error is introduced since we plan to evaluate the impact of seven parameters on the outcomes. To mitigate this risk, we will only conduct univariate meta-regression analysis. Moreover, we will only confidently assume effect modification in cases where the p-value is <0.01 and we will only consider the possibility of effect modification if p<0.05.

Moreover, we plan to conduct the following sensitivity analyses:

(a) For analyses focusing on stable COPD, we will only include studies only included participants who did not have any exacerbations and had not received any oral corticosteroids for at least three months prior to sampling.
(b) For analyses focusing on exacerbations, we will only include studies only including patients with a clinical diagnosis of COPD confirmed by spirometry during stable state.
(c) For analyses focusing on exacerbations, we will only include studies providing data for only one exacerbation per participant.
(d) We will repeat meta-analyses using fixed-effect models.
(e) We will only include studies of low risk of bias.

Certainty of evidence

For each outcome, we will evaluate the quality of the body of evidence using GRADE methodology, which takes into consideration study limitations, consistency of the effect, imprecision, indirectness, publication bias, magnitude of effect, dose response and confounders likely minimising the effect[17].

Protocol deviations

We will conduct our systematic review according to this published protocol. Any deviations will be documented and justified in our final report.

Patient and Public Involvement

This systematic review protocol was developed without patient or public involvement.

Ethics and Dissemination

Ethical approval is not a requirement since no primary data will be collected.

The findings of this systematic review and meta-analysis will be presented in national and international scientific conferences. They will also be submitted for publication in high-impact peer reviewed journals. It is anticipated that our findings will facilitate future research and inform clinical practice.

For peer review only

Discussion

Respiratory viral infections represent a major unmet treatment need in both stable COPD and exacerbations. The planned meta-analyses will systemise the existing evidence on their prevalence, clinical burden and -in the case of exacerbations- diagnostic techniques.

The prevalence of viral infections in patients with COPD exacerbations has been evaluated in two previous systematic reviews[25,26]. Both had limitations. Only PubMed was searched, and the most recent literature search was conducted in March 2017. When comparing the studies included in each of these reviews, we can see that important studies have been missed. Moreover, there is no formal assessment of the risk of bias of the included studies. Finally, quantitative analyses did not evaluate the prevalence of specific viruses, but rather the presence of any virus. To our knowledge, the prevalence of viral infections in patients with stable COPD and the clinical implications of the presence of viruses in either stable COPD or exacerbations has not been previously assessed in a systematic review.

Main strengths of our SRs include the clinically pertinent review questions, thorough searches of the available literature and methodological rigour. The inclusion of several meta-regressionand sensitivity analyses will improve our confidence on the certainty our findings. Potential unavoidable limitations include the lack of data availability on less prevalent viruses (such as metapneumovirus, which may not be assessed in many of the available studies) and technical limitations of the available studies, such as lack of standardization of the molecular techniques used to identify and quantify respiratory viruses. To address technical heterogeneity, we will report on the performance characteristics of the molecular techniques used. Challenges are anticipated in pooling viral loads, as several studies use relative quantification processes with little or no normalisation. In the main analysis, evaluating the mean viral loads during stable COPD and exacerbations, only studies reporting viral copies will be included. In an additional analysis, evaluating the relation of viral loads between stable COPD and exacerbations, we will also include studies using the same relative quantification process for both disease states.

We expect that this work could stimulate clinical and translational research, facilitate the introduction of precision medicine interventions both for stable COPD and exacerbations and improve the outcomes of these patients.

References

1. GBD 2015 Chronic Respiratory Disease Collaborators. Global, regional, and national deaths, prevalence, disability-adjusted life years, and years lived with disability for chronic obstructive pulmonary disease and asthma, 1990-2015: a systematic analysis for the Global Burden of Disease Study 2015. Lancet Respir Med 2017;5:691-706.

For peer review only

2. GBD Causes of Death Collaborators. Global, regional, and national age-sex specific mortality for 264 causes of death, 1980-2016: a systematic analysis for the Global Burden of Disease Study 2016. Lancet 2017;390:1151-210.
3. Vogelmeier CF, Criner GJ, Martinez FJ, et al. Global Strategy for the Diagnosis, Management, and Prevention of Chronic Obstructive Lung Disease 2017 Report: GOLD Executive Summary. Eur Respir J 2017;49.
4. Gayle A, Dickinson S, Morris K, Poole C, Mathioudakis AG, Vestbo J. What is the impact of GOLD 2017 recommendations in primary care? - a descriptive study of patient classifications, treatment burden and costs. Int J Chron Obstruct Pulmon Dis 2018;13:3485-92.
5. Agusti A, Bel E, Thomas M, et al. Treatable traits: toward precision medicine of chronic airway diseases. Eur Respir J 2016;47:410-9.
6. Seemungal T, Harper-Owen R, Bhowmik A, et al. Respiratory viruses, symptoms, and inflammatory markers in acute exacerbations and stable chronic obstructive pulmonary disease. Am J Respir Crit Care Med 2001;164:1618-23.
7. Wilkinson TMA, Aris E, Bourne S, et al. A prospective, observational cohort study of the seasonal dynamics of airway pathogens in the aetiology of exacerbations in COPD. Thorax 2017;72:919-27.
8. Bafadhel M, McKenna S, Terry S, et al. Acute exacerbations of chronic obstructive pulmonary disease: identification of biologic clusters and their biomarkers. Am J Respir Crit Care Med 2011;184:662-71.
9. George SN, Garcha DS, Mackay AJ, et al. Human rhinovirus infection during naturally occurring COPD exacerbations. Eur Respir J 2014;44:87-96.
10. Papi A, Bellettato CM, Braccioni F, et al. Infections and airway inflammation in chronic obstructive pulmonary disease severe exacerbations. Am J Respir Crit Care Med 2006;173:1114-21.
11. Lee N, Chan PK, Hui DS, et al. Viral loads and duration of viral shedding in adult patients hospitalized with influenza. J Infect Dis 2009;200:492-500.
12. Quint JK, Donaldson GC, Goldring JJ, Baghai-Ravary R, Hurst JR, Wedzicha JA. Serum IP-10 as a biomarker of human rhinovirus infection at exacerbation of COPD. Chest 2010;137:812-22.
13. Casanova V, Sousa FH, Stevens C, Barlow PG. Antiviral therapeutic approaches for human rhinovirus infections. Future Virol 2018;13:505-18.
14. Tickoo M, Ruthazer R, Bardia A, et al. The effect of respiratory viral assay panel on antibiotic prescription patterns at discharge in adults admitted with mild to moderate acute exacerbation of COPD: a retrospective before- after study. BMC Pulm Med 2019;19:118.
15. Mathioudakis AG, Chatzimavridou-Grigoriadou V, Corlateanu A, Vestbo J. Procalcitonin to guide antibiotic administration in COPD exacerbations: a meta-analysis. Eur Respir Rev 2017;26:160073.
16. Higgins JPT. Cochrane Handbook for Systematic Reviews of Interventions. The Cochrane Collaboration. 2011.
17. Guyatt GH, Oxman AD, Vist GE, et al. GRADE: an emerging consensus on rating quality of evidence and strength of recommendations. BMJ 2008;336:924-6.
18. Shamseer L, Moher D, Clarke M, et al. Preferred reporting items for systematic review and meta-analysis protocols (PRISMA-P) 2015: elaboration and explanation. BMJ 2015;350:g7647.
19. Moher D, Liberati A, Tetzlaff J, Altman DG, Group P. Preferred reporting items for systematic reviews and meta-analyses: the PRISMA statement. J Clin Epidemiol 2009;62:1006-12.
20. Loh CH, Genese FA, Kannan KK, Lovings TM, Peters SP, Ohar JA. Spirometry in Hospitalized Patients with Acute Exacerbation of COPD Accurately Predicts Post Discharge Airflow Obstruction. Chronic Obstr Pulm Dis 2018;5:124-33.
21. Mathioudakis AG, Janner J, Moberg M, Alonso-Coello P, Vestbo J. A systematic evaluation of the diagnostic criteria for COPD and exacerbations used in randomised controlled trials on the management of COPD exacerbations. ERJ Open Res 2019;5:00136-2019.
22. Mathioudakis AG, Moberg M, Janner J, Alonso-Coello P, Vestbo J. Outcomes reported on the management of COPD exacerbations: a systematic survey of randomised controlled trials. ERJ Open Res 2019;5:00072-2019.
23. Hoy D, Brooks P, Woolf A, et al. Assessing risk of bias in prevalence studies: modification of an existing tool and evidence of interrater agreement. J Clin Epidemiol 2012;65:934-9.

For peer review only

24. Wells GA, Shea B, O'Connell D, et al. The Newcastle-Ottawa Scale (NOS) for assessing the quality if nonrandomized studies in meta-analyses. (Accessed 18/08/2019, at: http://www.ohri.ca/programs/clinical_epidemiology/oxford.htm).

25. Jafarinejad H, Moghoofei M, Mostafaei S, Salimian J, Azimzadeh Jamalkandi S, Ahmadi A. Worldwide prevalence of viral infection in AECOPD patients: A meta-analysis. Microb Pathog 2017;113:190-6.

26. Wu X, Chen D, Gu X, Su X, Song Y, Shi Y. Prevalence and risk of viral infection in patients with acute exacerbation of chronic obstructive pulmonary disease: a meta-analysis. Mol Biol Rep 2014;41:4743-51.

Author E-mails:

Anastasia M. Kefala: anastasia.m.kefala@gmail.com,

Rebecca Fortescue: rnormans@sgul.ac.uk,

Gioulinta S. Alimani: juli.alimani95@gmail.com,

Prodromos Kanavidis: makis.k@gmail.com,

Melissa Jane McDonnell: melissajanemcdonnell@gmail.com,

Emmanouil Magiorkinis: emmanouil.magiorkinis@gmail.com,

Spyridon Megremis: spyridon.megremis@manchester.ac.uk,

Dimitrios Paraskevis: dparask@med.uoa.gr,

Chrysa Voyiatzaki: cvoyiatz@uniwa.gr,

Georgios A. Mathioudakis: geoamath@gmail.com,

Effie Papageorgiou: efipapag@uniwa.gr,

Nikolaos G. Papadopoulos: ngpallergy@gmail.com,

Jørgen Vestbo: Jorgen.Vestbo@manchester.ac.uk,

Apostolos Beloukas: abeloukas@uniwa.gr,

Alexander G. Mathioudakis: Alexander.Mathioudakis@manchester.ac.uk.

For peer review only

Online Appendix

Title: Prevalence and clinical implications of respiratory viruses in stable chronic obstructive pulmonary disease (COPD) and exacerbations: A systematic review and meta-analysis protocol.

Authors: Anastasia M. Kefala, Rebecca Fortescue, Gioulinta S. Alimani, Prodromos Kanavidis, Melissa Jane McDonnell, Emmanouil Magiorkinis, Dimitrios Paraskevis, Chrysa Voyiatzaki, Georgios A. Mathioudakis, Nikolaos G. Papadopoulos, Jørgen Vestbo, Apostolos Beloukas, Alexander G. Mathioudakis.

Search Strategy - Medline, PubMed & Cochrane Library

#1 Chronic Obstructive Pulmonary Disease [MH]

#2 Lung Diseases, Obstructive [MH:NOEXP]

#3 Emphysema [MH]

#4 Chronic Bronchitis [MH]

#5 COPD [tiab]

#6 COAD [tiab]

#7 "Chronic Bronchitis" [tiab]

#8 Emphysema [tiab]

#9 Obstructive[ti]

#10 (Pulmonary OR Respiratory OR Airway OR Airflow OR Lung)[ti]

#11 #9 AND #10

#12 AECOPD[tiab]

#13 IECOPD[tiab]

#14 #1 OR #2 OR #3 OR #4 OR #5 OR #6 OR #7 OR #8 OR #11 OR #12 OR #13

#15 Viruses[MH]

#16 Influenza, Human[MH]

#17 Rhinovirus[MH]

#18 Respiratory Syncytial Viruses[MH]

#19 Coronavirus[MH]

#20 Paramyxoviridae Infections[MH]

#21 Orthomyxoviridae[MH]

#22 Adenoviridae [MH]

For peer review only

#23 Picornaviridae[MH]

#24 Metapneumovirus[MH]

#25 Enterovirus[MH]

#26 Cytomegalovirus[MH]

#27 Herpesvirus 3, Human[MH]

#28 Virus*[tiab]

#29 Viral*[tiab]

#30 Influenza*[tiab]

#31 Rhinovir*[tiab]

#32 Respiratory Syncytial Vir*[tiab]

#33 Coronavir*[tiab]

#34 Paramyxovir*[tiab]

#35 Orthomyxovir*[tiab]

#36 Adenovir*[tiab]

#37 Picornavir*[tiab]

#38 Metapneumov*[tiab]

#39 vzv[tiab]

#40 varicella[tiab]

#41 Enterovir*[tiab]

#42 Parainfluenza[tiab]

#43 Echovir*[tiab]

#44 #15 or #16 or #17 or #18 or #19 or #20 or #21 or #22 or #23 or #24 or #25 or #26 or #27 or #28 or #29 or #30 or #31 or #32 or #33 or #34 or #35 or #36 or #37 or #38 or #39 or #40 or #41 or #42 or #43

#45 Animals[mh] not (humans[mh])

#46 (child[mh]or (adolescent[mh])) not (adult[mh])

#47 editorial[pulication type]

#48 review[publication type] not (systematic review [publication type])

#49 #14 and #44

#50 #49 NOT (#45 or #46 or #47 or #48)

For peer review only

Search Strategy: EMBASE

1 exp Chronic Obstructive Pulmonary Disease/

2 exp Lung diseases, obstructive/

3 exp Chronic bronchitis/

4 exp emphysema/

5 COPD.tw.

6 COAD.tw.

7 (chronic adj2 bronchit$).tw.

8 (obstructive adj3 (pulmonary or lung$ or airway$ or airflow$ or bronch$ or respirat$)).tw.

9 AECOPD.tw

10 IECOPD.tw

11 ECOPD.tw

12 1 or 2 or 3 or 4 or 5 or 6 or 7 or 8 or 9 or 10 or 11

13 exp virus/

14 exp influenza/

15 exp rhinovirus/

16 exp Respiratory Syncytial Virus/

17 exp Coronavirus/

18 exp Paramyxoviridae Infections/

19 exp Orthomyxoviridae/

20 exp Adenoviridae/

21 exp Picornaviridae/

22 exp Metapneumovirus/

23 exp Enterovirus/

24 exp Cytomegalovirus/

25 exp bocavirus/

26 virus$.tw.

27 viral$.tw.

28 Influenza$.tw.

29 (("haemophilus influenzae" or "h. influenza") not influenza).tw.

For peer review only

30 28 not 29

31 Rhinovir$.tw.

32 Respiratory Syncytial Vir$.tw.

33 Coronavir$.tw.

34 Paramyxovir$.tw.

35 Orthomyxovir$.tw.

36 Adenovir$.tw.

37 Picornavir$.tw.

38 Metapneumov$.tw.

39 Enterovir$.tw.

40 Parainfluenza.tw.

41 Echovir$.tw.

42 Bocavir$.tw.

43 vzv.tw.

44 varicella.tw.

45 13 or 14 or 15 or 16 or 17 or 18 or 19 or 20 or 21 or 22 or 23 or 24 or 25 or 26 or 27 or 30 or 31 or 32 or 33 or 34 or 35 or 36 or 37 or 38 or 39 or 40 or 41 or 42 or 43 or 44

46 exp animals/ not exp humans/

47 (exp child/ or exp adolescent/) not exp adult/

48 exp editorial/ or (exp review/ not (exp systematic review/))

49 12 and 45

50 49 not (46 or 47 or 48)

For peer review only

Data extraction: Variables to be captured.

Study characteristics - General: study ID, study/cohort name, study registration number, full references of all citations referring to the study, year of publication, country(-ies), continent(s), number of recruiting centres, study design, study setting.

Description of the study population: Stable COPD or exacerbations, general or specific population tested, total number of participants evaluated during stable disease, total number of participants evaluated during exacerbation, age, sex, smoking history, sampling period, %sampled during the flu season [October-May], spirometric severity of COPD, history of exacerbations, respiratory symptoms [mMRC, CAT], use of ICS (%), severity of the index exacerbation, >1 exacerbation allowed per participant.

Risk of bias assessment: The relevant risk of bias tool(s) will be completed for each included study.

Respiratory viruses: Type of sample, number of viruses evaluated, number of patients testing positive for any virus, number of patients tested for any virus, number of patients testing positive for more than one virus, number of patients testing positive for each virus type or subtype tested, number of patients that were tested in each case, data on the viral loads of each virus, assay used (commercial versus in-house), performance characteristics of the assays used.

Clinical outcomes: Data on each of the selected outcome measures will be extracted. These will include a detailed description of the definition of the outcome, the effect estimates and confidence intervals, as well as the timing of every measurement. Outcome measures in relevant subgroups of participants will also be extracted.

Data collected during stable disease versus exacerbations will be clearly separated.

For peer review only

Prevalence and clinical implications of respiratory viruses in stable chronic obstructive pulmonary disease (COPD) and exacerbations: A systematic review and meta-analysis protocol.

Anastasia M. Kefala, Rebecca Fortescue, Gioulinta S. Alimani, Prodromos Kanavidis, Melissa Jane McDonnell, Emmanouil Magiorkinis, Spyridon Megremis, Dimitrios Paraskevis, Chrysa Voyiatzaki, Georgios A. Mathioudakis, Effie Papageorgiou, Nikolaos G. Papadopoulos, Jørgen Vestbo, Apostolos Beloukas, Alexander G. Mathioudakis.

PRISMA-P 2015 Checklist

This checklist has been adapted for use with protocol submissions to *Systematic Reviews* from Table 3 in Moher D et al: Preferred reporting items for systematic review and meta-analysis protocols (PRISMA-P) 2015 statement. *Systematic Reviews* 2015 **4**:1

Section/topic	#	Checklist item	Information reported: Yes	Information reported: No	Line number(s)
**ADMINISTRATIVE INFORMATION**					
**Title**					
Identification	1a	Identify the report as a protocol of a systematic review	☒	☐	1-2
Update	1b	If the protocol is for an update of a previous systematic review, identify as such	☐	☒	N/A
**Registration**	2	If registered, provide the name of the registry (e.g., PROSPERO) and registration number in the Abstract	☒	☐	79-80
**-Authors**					
Contact	3a	Provide name, institutional affiliation, and e-mail address of all protocol authors; provide physical mailing address of corresponding author	☒	☐	4-37, 379-394
Contributions	3b	Describe contributions of protocol authors and identify the guarantor of the review	☒	☐	41-44
**Amendments**	4	If the protocol represents an amendment of a previously completed or published protocol, identify as such and list changes; otherwise, state plan for documenting important protocol amendments	☒	☐	280-282
**Support**					
Sources	5a	Indicate sources of financial or other support for the review	☒	☐	45-47
Sponsor	5b	Provide name for the review funder and/or sponsor	☐	☒	N/A

For peer review only

Section/topic	#	Checklist item	Information reported		Line number(s)
			Yes	No	
Role of sponsor/funder	5c	Describe roles of funder(s), sponsor(s), and/or institution(s), if any, in developing the protocol	☐	☒	N/A
**INTRODUCTION**					
**Rationale**	6	Describe the rationale for the review in the context of what is already known	☒	☐	95-131
**Objectives**	7	Provide an explicit statement of the question(s) the review will address with reference to participants, interventions, comparators, and outcomes (PICO)	☒	☐	132-1143, Methods
**METHODS**					
**Eligibility criteria**	8	Specify the study characteristics (e.g., PICO, study design, setting, time frame) and report characteristics (e.g., years considered, language, publication status) to be used as criteria for eligibility for the review	☒	☐	152-173
**Information sources**	9	Describe all intended information sources (e.g., electronic databases, contact with study authors, trial registers, or other grey literature sources) with planned dates of coverage	☒	☐	207-216
**Search strategy**	10	Present draft of search strategy to be used for at least one electronic database, including planned limits, such that it could be repeated	☒	☐	207-216, online appendix
STUDY RECORDS					
Data management	11a	Describe the mechanism(s) that will be used to manage records and data throughout the review	☒	☐	217-221
Selection process	11b	State the process that will be used for selecting studies (e.g., two independent reviewers) through each phase of the review (i.e., screening, eligibility, and inclusion in meta-analysis)	☒	☐	217-221
Data collection process	11c	Describe planned method of extracting data from reports (e.g., piloting forms, done independently, in duplicate), any processes for obtaining and confirming data from investigators	☒	☐	222-228
**Data items**	12	List and define all variables for which data will be sought (e.g., PICO items, funding sources), any pre-planned data assumptions and simplifications	☒	☐	222-228
**Outcomes and prioritization**	13	List and define all outcomes for which data will be sought, including prioritization of main and additional outcomes, with rationale	☒	☐	174-206
**Risk of bias in individual studies**	14	Describe anticipated methods for assessing risk of bias of individual studies, including whether this will be done at the outcome or study level, or both; state how this information will be used in data synthesis	☒	☐	229-241
DATA					
**Synthesis**	15a	Describe criteria under which study data will be quantitatively synthesized	☒	☐	242-251
	15b	If data are appropriate for quantitative synthesis, describe planned summary measures, methods of handling data, and methods of combining data from studies, including any planned exploration of consistency (e.g., I^2, Kendall's tau)	☒	☐	243-245

For peer review only

Section/topic	#	Checklist item	Information reported Yes	No	Line number(s)
	15c	Describe any proposed additional analyses (e.g., sensitivity or subgroup analyses, meta-regression)	☒	☐	252-275
	15d	If quantitative synthesis is not appropriate, describe the type of summary planned	☐	☒	N/A
**Meta-bias(es)**	16	Specify any planned assessment of meta-bias(es) (e.g., publication bias across studies, selective reporting within studies)	☒	☐	240-241
**Confidence in cumulative evidence**	17	Describe how the strength of the body of evidence will be assessed (e.g., GRADE)	☒	☐	276-279

For peer review only
